# Skin-limited Langerhans cell histiocytosis in an adult presenting as isolated, eroded, “kissing” intergluteal plaques

**DOI:** 10.1016/j.jdcr.2023.09.019

**Published:** 2023-10-01

**Authors:** Molly E. Kuo, Andrew Schuler, Asra Ahmed, Emily Smith, Frank Wang

**Affiliations:** a Medical Scientist Training Program, University of Michigan Medical School, Ann Arbor, Michigan; b Department of Dermatology, University of Michigan, Ann Arbor, Michigan; c Section of Dermatopathology, Department of Pathology, University of Michigan, Ann Arbor, Michigan; d Division of Hematology and Oncology, Department of Internal Medicine, University of Michigan, Ann Arbor, Michigan

**Keywords:** intergluteal cleft erosion, Langerhans cell histiocytosis

## Introduction

Langerhans cell histiocytosis (LCH) is characterized by clonal proliferation of histiocytes accumulating in a variety of organs, most often the skin, bone, lungs, and pituitary gland.^1^^,^^2^ Although LCH is most commonly observed in children, it can rarely affect adults.^3^ While LCH can be skin-limited, most patients with cutaneous disease have multisystem involvement, which is associated with poorer prognosis.^2^ We report a case of an adult man with a history of liver transplant presenting with an intergluteal rash that was diagnosed as cutaneous LCH.

## Case report

A 63-year-old man presented to our clinic with a 6-month history of a waxing and waning, tender rash affecting the intergluteal cleft. He had previously applied topical terbinafine 1%, tolnaftate 1%, clotrimazole 1%, triple antibiotic cream, and hydrocortisone 1% without resolution of the rash. His medical history was remarkable for primary sclerosing cholangitis status post liver transplant 5 years earlier, immunosuppression with mycophenolate mofetil and oral tacrolimus, and melanoma in situ on the left heel 3 years earlier.

**Fig 1:**
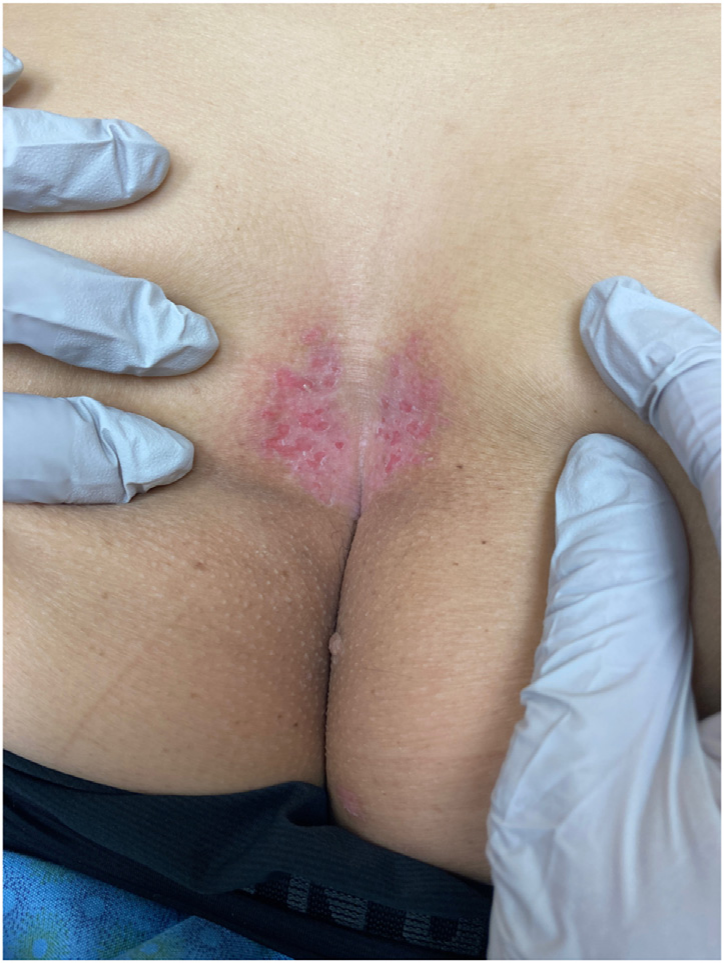
*Pink* plaques with numerous “punched out” erosions, symmetrically affecting the superior intergluteal cleft and bilateral buttocks in a “kissing” fashion.

Physical exam revealed pink plaques with numerous coalescing “punched out” erosions, symmetrically affecting the superior intergluteal cleft and bilateral buttocks in a “kissing” fashion (Fig 1). No satellite lesions were noted. Differential diagnosis included herpetic infection, extramammary Paget disease, intertrigo, and allergic or irritant contact dermatitis.

**Fig 2:**
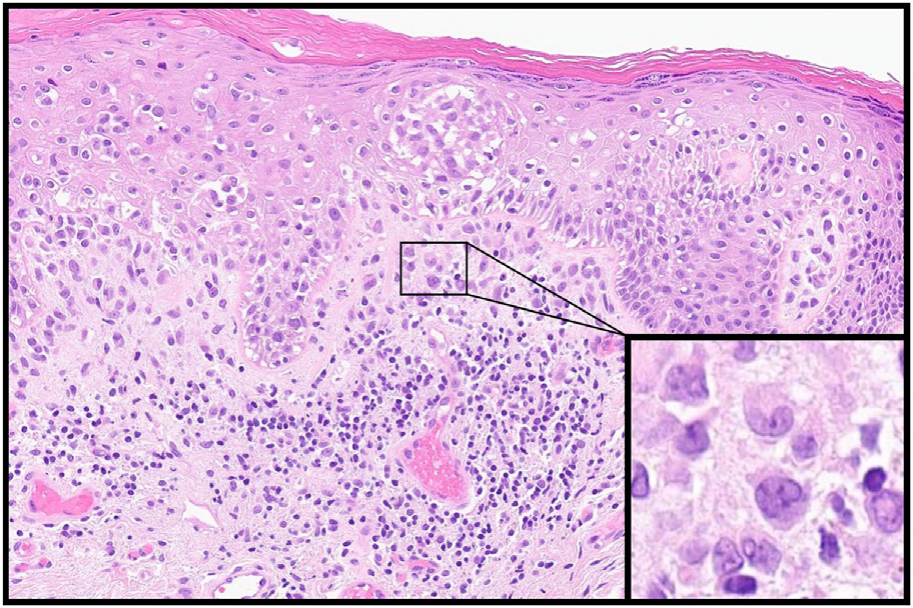
Histopathologic examination revealed a dense superficial dermal infiltrate of histiocytes with reniform nuclei, lymphocytes, and occasional eosinophils. Inset shows cells with reniform nuclei and eosinophilic cytoplasm, suggestive of Langerhans cells.

**Fig 3:**
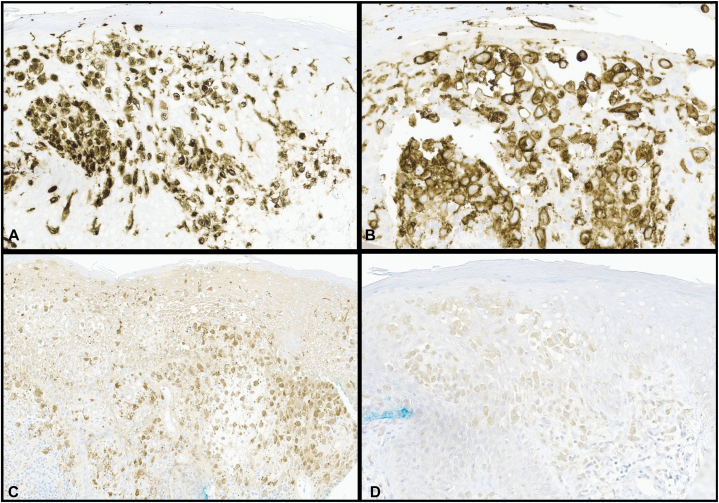
Immunohistochemistry revealed a histiocytic infiltrate staining positively for (**A**), langerin, (**B**), CD1a, (**C**), S100, (**D**), and *BRAF* V600E mutation, consistent with Langerhans cell histiocytosis.

**Fig 4:**
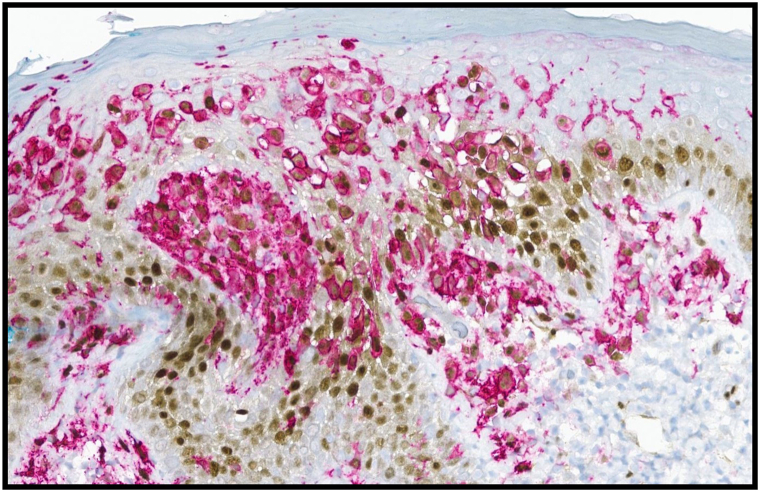
Additional immunohistochemistry with cyclin D1 (*brown* nuclear staining) / CD1a (*red* membranous staining) dual stain revealed that the Langerhans cells were positive for cyclin D1, supporting a diagnosis of Langerhans cell histiocytosis.

Initial work-up included a viral swab, which was negative for herpes simplex virus and varicella zoster virus, and a punch biopsy, with histopathologic examination revealing epidermal erosions and a dense infiltrate of histiocytes with reniform nuclei and nuclear grooves in the papillary dermis (Fig 2). Scattered lymphocytes and eosinophils were observed. Immunostaining demonstrated that the histiocytes were positive for langerin, CD1a, S100, and *BRAF* V600E mutation (Fig 3). They were also positive for cyclin D1 expression on cyclin D1/CD1a dual staining, which has demonstrated value in discriminating neoplastic from reactive Langerhans cells (Fig 4).^4^

Considering the clinical and histopathologic findings, a diagnosis of LCH was rendered, and additional workup was initiated to exclude systemic disease. The patient reported feeling well and denied headache, bone pain, shortness of breath, polydipsia, polyuria, abdominal pain, or other symptoms. A complete blood count, comprehensive metabolic panel, and monoclonal gammopathy evaluation were unremarkable.

Subsequently, the patient underwent a full-body positron emission tomography scan, which showed mild hypermetabolism within the intergluteal cleft consistent with cutaneous LCH, and no evidence of systemic disease. Evaluation by hematology/oncology confirmed that his presentation was consistent with skin-limited LCH. The patient’s intergluteal rash resolved with consistent usage of triamcinolone 0.1% ointment in a burst and taper fashion.

## Discussion

We report an unusual case of an adult man who presented with an eroded, tender, intergluteal rash diagnosed as LCH, based on histopathologic analysis. Further workup, including full-body positron emission tomography scan and laboratory tests, excluded systemic involvement, allowing us to render a diagnosis of skin-limited LCH.

LCH most commonly affects children with an estimated incidence of 4 to 5 cases/million/year.^2^^,^^5^ When affecting the skin, LCH often presents as pinpoint erythematous, purpuric or skin-colored pustules or papules in the scalp and body folds, mimicking seborrheic dermatitis, eczema, or diaper dermatitis.^2^ Given the propensity to involve multiple organ systems and its associated morbidity and potential mortality, LCH is a crucial diagnosis to consider in children.^2^

In adults, LCH is much less common with an estimated incidence of 1 to 1.5 cases/million/year.^3^^,^^6^ Importantly, given its rarity, adult-onset LCH needs to be studied further and may not be considered in the differential diagnosis of dermatoses involving the scalp and intertriginous sites.^7^ In adult-onset cases, multisystem LCH is estimated to involve the skin in 20% to 50% of cases, whereas skin-limited LCH is far less common.^3^^,^^7^^,^^8^ Indeed, skin-limited presentations are estimated to comprise only 5% to 10% of adult-onset LCH cases.^3^^,^^7^^,^^8^

Presentation in adults is similar to that in children, with erythematous to purpuric papules, sometimes with vesiculation or crusting.^3^ The chest, back, abdomen, limbs, scalp, and groin are most commonly affected, whereas involvement of the oral mucosa, genitalia, or perianal region is less common.^3^^,^^9^ In the gluteal area, the differential diagnosis includes herpetic infection, candidal or dermatophyte infection, extramammary Paget disease, intertrigo, lichen sclerosus, granular parakeratosis, psoriasis, Hailey-Hailey disease, and contact dermatitis.

Diagnosis can be rendered with a biopsy demonstrating representative histopathology.^3^ The pathogenesis of LCH involves activation of the mitogen-activated protein kinase signaling pathway, and therefore, testing for the *BRAF* V600E mutation, seen in 57% of LCH lesions, is recommended.^1^^,^^3^ If the diagnosis of cutaneous LCH is made, full-body fluorodeoxyglucose-positron emission tomography/computed tomography imaging is recommended to determine the extent of systemic involvement.^3^ Laboratory tests should include a complete blood count, comprehensive metabolic panel, and monoclonal gammopathy evaluation.

The treatment for skin-limited LCH depends on the severity of the presentation and typically involves topical corticosteroids or oral therapy, including hydroxyurea and low-dose methotrexate.^3^ Refractory lesions may respond to imiquimod, nitrogen mustard, irradiation, systemic chemotherapy, or targeted systemic agents, such as vemurafenib.^3^ Close surveillance, including complete skin exams at each visit, is recommended for patients with skin-limited LCH.^3^

Interestingly, when LCH affects the liver, it most commonly causes sclerosing cholangitis.^10^ Our patient was diagnosed with this condition years before his LCH diagnosis, raising the question of whether LCH may have been responsible for the biliary dysfunction. However, no Langerhans cells or positive CD1a and S100 staining was present on histopathologic analysis of his liver biopsy, so the connection is unlikely. Furthermore, our patient had a history of liver transplant and immunosuppression. To the authors’ knowledge, there is not an established connection between these conditions and the development of LCH. In fact, organ transplantation and immunosuppressive agents can be used to treat severe LCH in certain situations.^3^

Our case highlights the importance of considering LCH as a diagnostic possibility when evaluating adults with a long-standing gluteal or intertriginous rash with erosions/ulcerations. In such cases, clinicians should have a low threshold to obtain a biopsy. Rendering the diagnosis is particularly important, because skin involvement of LCH in adults is commonly associated with multisystem disease, necessitating a complete work-up. Despite the rarity of adult-onset LCH, clinicians should be aware of isolated presentations in the gluteal region to avoid potential morbidity and mortality from delayed diagnosis.

## Conflicts of interest

None disclosed.
